# Production Before Comprehension in the Emergence of Transitive Constructions in Dutch Child Language

**DOI:** 10.3389/fpsyg.2020.546495

**Published:** 2020-10-26

**Authors:** Gisi Cannizzaro, Petra Hendriks

**Affiliations:** Center for Language and Cognition Groningen, University of Groningen, Groningen, Netherlands

**Keywords:** animacy, child language, Dutch, eye-tracking, language acquisition, production-comprehension asymmetry, transitive constructions, word order

## Abstract

Although 2-year-old English- or Dutch-speaking children tend to use correct subject-object word order in their own utterances, they appear to make a substantial number of word order errors in their comprehension of other people’s utterances. This pattern of adult-like production but poor comprehension is challenging for linguistic theory. While most approaches to language acquisition explain this pattern from extra-linguistic factors such as task demands, the constraint-based approach Optimality Theory predicts this asymmetry between production and comprehension to arise as a result of the linguistic competition between constraints on word order and animacy. This study tests this prediction by investigating how children’s comprehension and production of word order in transitive constructions develop, and to what degree their comprehension and production are influenced by animacy. Two- and three-year-old Dutch speaking children (*n* = 32) and adult controls (*n* = 41) were tested on their comprehension and production of simple transitive sentences, in which the animacy of the grammatical subject and object were manipulated. Comprehension was tested in a picture selection task and a preferential looking task, and production was tested in a parallel sentence elicitation task. Children’s comprehension of transitive sentences in the picture selection task was found to be less accurate than their production of the same sentences in the sentence elicitation task. Their eye gaze in the minimally demanding preferential looking task did not reveal a more advanced understanding of these sentences. In comprehension, children’s response accuracy, and to a lesser extent their eye gaze, was influenced by the animacy of subject and object, providing evidence that their poor comprehension is due to the competition between word order and animacy, as predicted by the constraint-based approach. In contrast, animacy may have a facilitating effect on children’s production of transitive sentences. These findings suggest that the mature form and meaning of a transitive construction are not acquired together. Rather, the form-meaning pairings of transitive constructions seem to arise gradually as the by-product of acquiring the constraint ranking of the grammar. This leads to the gradual alignment of forms and meanings in child language and hence to the emergence of linguistic constructions.

## Introduction

A central task for children acquiring their native language is learning how the language expresses who is doing what to whom. This is marked by word order in English: in active transitive sentences, the first noun phrase is the subject and the second one is the direct object. For example, in the sentence *The car is pushing the cow* the first noun phrase *the car* is the grammatical subject (and hence the agent performing the action), and the second noun phrase *the cow* is the direct object (and hence the patient that is acted upon).

English-speaking children between the ages of one and two have been found to already be sensitive to the word order of English (e.g., Hirsh-Pasek and Golinkoff, 1996; Gertner et al., 2006; Candan et al., 2012). However, 2-year-old English-speaking children still make a large number of word order errors in their interpretation of simple transitive sentences when word order conflicts with other potential cues for interpretation (Chapman and Miller, 1975; McClellan et al., 1986; Thal and Flores, 2001; Chan et al., 2009). Specifically, young children show large variability in their interpretations when other cues are present. For example, they may interpret the most animate noun phrase (e.g., *the cow* in the example above) as the subject of the sentence and the agent performing the action, rather than the first-mentioned noun phrase (Chapman and Miller, 1975).

Contrasting with their variable interpretations, 2-year-old English-speaking children appear to be surprisingly consistent in their use of word order in their own utterances. Their production of simple transitive sentences is largely adult-like, regardless of the animacy properties of the noun phrases or the probability of the events (Chapman and Miller, 1975; Angiolillo and Goldin-Meadow, 1982; McClellan et al., 1986). Thus, in the acquisition of transitive constructions, 2-year-olds’ production seems to be ahead of their comprehension.

This raises the question how poor comprehension coupled with adult-like production of transitive sentences should be explained. Several studies (e.g., Bates et al., 1995) have dismissed the finding of poor comprehension as a confound of the complex task demands of the comprehension tasks used, which may underestimate children’s knowledge of word order. At the same time, children’s knowledge of word order may be overestimated in production tasks, according to Bates et al. (1995). Children’s production may only appear to be adult-like because they are simply repeating forms that they have heard before and have memorized. Consequently, the observation of a production-comprehension asymmetry may merely be an artifact of the experimental tasks used. On the other hand, it is possible that there is an actual asymmetry between production and comprehension as children acquire word order. If so, this will have important implications for our view of the emergence of transitive constructions in child language, given that most linguistic theories assume that production and comprehension of a construction develop in parallel.

This paper addresses the question of how children’s comprehension and production of word order in transitive constructions develop, and to what degree their comprehension and production of these constructions are influenced by animacy. It focuses on Dutch. To answer these questions, young Dutch-speaking children are tested on their comprehension and production of simple transitive sentences in which the animacy of the subject and the object are manipulated. Comprehension is tested in a picture selection task and a minimally demanding preferential looking task in which children’s eye gaze during sentence interpretation is tracked. Production is tested in a parallel sentence elicitation task. Before presenting this experiment in Section “Experiment,” we will review relevant previous experimental findings as well as the conflicting theoretical perspectives on the acquisition of transitive constructions.

## Background

Children acquiring English have been found to already be sensitive to word order from an early age (e.g., de Villiers and de Villiers, 1973; Hirsh-Pasek and Golinkoff, 1996; Gertner et al., 2006; Candan et al., 2012). For example, using the intermodal preferential looking paradigm, Hirsh-Pasek and Golinkoff (1996) showed that English-speaking children are sensitive to the order of subject and object already at the age of 17 months old. In this paradigm, children see two videos showing two events presented side-by-side on television monitors, while a sentence is played, for example *Where is Cookie Monster washing Big Bird?* All sentences contained a reversible verb and two animate noun phrases. Therefore, word order was the only cue for interpretation. The matching video showed the event with the first noun phrase as the agent, and the non-matching video showed the event with the second noun phrase as the agent. The rationale for this task is that children pay more attention to the video that matches what they are hearing. Because the children in their study looked more at the matching video than at the non-matching video, Hirsh-Pasek and Golinkoff concluded that the children were guided by word order in their interpretation of the sentence. Later studies also using preferential looking tasks confirmed these results (e.g., Candan et al., 2012; and, testing novel verbs, Gertner et al., 2006). This suggests that word order is available as a cue to sentence interpretation before age 2 in English.

However, studies in which word order was not the only cue for interpretation found that 2-year-old English-speaking children still make a large number of word order errors with simple transitive sentences and show variability in their interpretation (Chapman and Miller, 1975; McClellan et al., 1986; Thal and Flores, 2001; Chan et al., 2009). For example, in Chapman and Miller’s (1975) act-out study with English-speaking children between the ages of 1;8 and 2;8, children’s interpretations as acted out with toys were largely correct when the subject was animate and the object was inanimate. However, when the subject was inanimate and the object was animate, for example in the sentence *The boat is pulling the girl*, children’s interpretations were correct only about half of the time. The incorrect responses revealed that the children interpreted the animate noun phrase as the subject, rather than the first noun phrase. In addition to this comprehension task, Chapman and Miller’s children carried out a parallel production task in which they had to describe events that the experimenter acted out with toys. The same children who showed poor comprehension now produced utterances with correct word order in over 80% of cases, without much variation between the animacy conditions. From this, Chapman and Miller concluded that young English-speaking children’s competence of subject-object word order is less advanced and different in comprehension than in production. McClellan et al. (1986) found a similar asymmetry between comprehension and production in an act-out task with English-speaking 2-year-olds, although their children did not base their comprehension on animacy but instead selected the most probable event as the sentence meaning.

This pattern in English has also been found in Dutch. The 2-year-old Dutch-speaking children Cannizzaro (2012) tested with an act-out task (in an experiment with animals and vehicles and another experiment with humans and vehicles) also performed more poorly in comprehension (59 and 62% correct) than in production (85 and 81% correct). The 3-year-olds in her study performed overwhelmingly well in comprehension (92 and 83% correct) as well as in production (100 and 95% correct). An animacy effect on comprehension was found when 3-year-olds interpreted sentences with humans, but not with animals; with 2-year-olds, no animacy effects were found at all.

However, later studies criticized Chapman and Miller’s (1975) conclusion and argued that the poor comprehension that the children exhibited must be due to experimental artifacts (e.g., Bates et al., 1995). Most studies demonstrating poor comprehension of simple transitive sentences used act-out tasks, which require children to act out their interpretation of a heard sentence with toys. However, act-out tasks are cognitively demanding and have been shown to result in response biases (Goodluck, 1996). Thus, the studies using act-out tasks may have underestimated children’s knowledge of word order in transitive constructions (Bates et al., 1995). To our knowledge, no studies have been carried out to test the same children’s comprehension and production of simple transitive sentences using a less demanding comprehension task.

A large body of research on children’s development of transitive constructions in recent years has focused on an issue that is largely independent of the existence of a production-comprehension asymmetry, namely whether children’s early knowledge of transitive constructions is abstract and rule-like, as is assumed in parameter-based or generativist approaches, or driven by concrete items and gradual, as is assumed in usage-based or constructivist approaches. According to parameter-based approaches (e.g., Guasti, 2002; Franck et al., 2013), the abstract universal principles of language are innately specified, and children only have to acquire the language-specific parameter settings. As soon as the relevant parameters for subject-object word order, such as the head direction parameter, are set on the basis of specific language input, their production of word order will be adult-like. Because syntactic representations form the input to the interpretation module, children’s comprehension of word order is then expected to be adult-like too. Thus, parameter-based approaches do not predict any asymmetries between production and comprehension in child language, apart from performance errors such as those due to the demands of the experimental tasks (Grimm et al., 2011, p. 2).

According to usage-based approaches (e.g., Abbot-Smith and Tomasello, 2006; Ambridge and Lieven, 2015), children first acquire concrete representations that are tied to specific words and their meanings on the basis of the language input they receive. From these rote-learned form-meaning mappings, using general cognitive skills children then develop lexically specific ‘slot-and-frame’ schemas such as for transitive sentences. These schemas may initially still be quite fragile, but gradually develop into fully abstract adult-like constructions. Although schemas represent syntactic as well as semantic knowledge and could thus result in a parallel development of production and comprehension, usage-based approaches also assign a central role to cognitive processes and heuristics. Theakston et al. (2012, p. 122) speculate that animacy may explain why children’s early knowledge of transitive constructions in comprehension around age 2;0 precedes their knowledge in production, which is the inverse of the asymmetry discussed above. Ambridge and Lieven (2011, p. 236) suggest that cognitive processes may explain this comprehension-before-production pattern, referring to a connectionist simulation study by Chang et al. (2006). However, as this simulation study modeled comprehension as predicting the next word in the sentence, it is doubtful whether this connectionist simulation study accurately reflects children’s comprehension processes. Also, it has yet to be determined how heuristics and cognitive processes can explain this early comprehension-before-production asymmetry and at the same time explain the later production-before-comprehension asymmetry that is the focus of this study. As it is still an open question in usage-based linguistics to which extent children draw on the same heuristics in production and comprehension (Lieven, 2016, p. 354), usage-based approaches do not, in and of themselves, make *a priori* predictions about where asymmetries occur in child language.

Contrasting with these two approaches, the constraint-based approach Optimality Theory (Prince and Smolensky, 1993/2004) predicts that for certain linguistic forms comprehension precedes production (Smolensky, 1996), whereas for other forms production precedes comprehension (Hendriks, 2016), depending on the linguistic constraints involved. According to Optimality Theory, the realization and interpretation of words and sentences follows from the interplay between conflicting constraints of various sorts. These constraints express general tendencies in the language that can be in conflict with one another and can be violated in order to satisfy other, stronger, constraints. The constraints may either be innately specified or functionally motivated (see Lestrade et al., 2016; van de Weijer, 2019, for discussion). The optimal output is the output that satisfies the constraints of the grammar best, and is the realized output (i.e., the produced form or selected interpretation). Optimality Theory models production and comprehension as different directions of optimization based on the same constraints. In production, the input is a meaning and the output is the optimal form from a set of potential forms for expressing this meaning. In comprehension, the input is a form and the output is the optimal meaning from a set of potential meanings for that form. Asymmetries between production and comprehension arise from the different directions of optimization in production and comprehension (Smolensky, 1996; Hendriks, 2014, 2016). Because the constraints are output-oriented and either evaluate the output in relation to other outputs (markedness constraints), or evaluate the output in relation to the input (faithfulness constraints), the same output-oriented constraints can have different effects in production and comprehension, as the output differs in production and comprehension. For example, a constraint evaluating meanings will only have an effect in comprehension, when the output is a meaning, but not in production, when the output is a form. This is illustrated below.

To account for word order phenomena within Optimality Theory, two violable constraints have been argued to play an essential role, namely a constraint pertaining to word order and a constraint pertaining to animacy. The word order constraint requires subjects to linearly precede objects (cf. Greenberg’s language universal 1, Greenberg, 1966, p. 61) and has been motivated independently to account for the interaction between word order, case, and animacy in sentence comprehension in German and Dutch (de Hoop and Lamers, 2006); patterns of word order variation and word order freezing in various languages (e.g., Lee, 2001; Bouma and Hendriks, 2012); and the acquisition of wh-questions in Dutch and German (Schouwenaars et al., 2014, 2018). The second constraint is an animacy constraint that requires the subject to be animate and the object to be inanimate (Aissen, 2003), or, in a slightly stronger formulation, requires the subject to be higher in animacy than the object on a scale of animacy ranking humans above animals, and animals above inanimate entities (de Hoop and Lamers, 2006; de Hoop and Malchukov, 2008; de Swart, 2011; de Swart and van Bergen, 2019).

This relational animacy constraint, relating the animacy of subject and object in comparison to one another, must be distinguished from the inherent animacy bias that is familiar from the sentence processing literature and holds that animate entities are conceptually more accessible than inanimate entities (e.g., Bock and Warren, 1985; Branigan et al., 2008). The animacy constraint, but not the inherent animacy bias, plays a role in Optimality Theory accounts of word order phenomena and is functionally grounded in the need to distinguish the subject from the object when the sentence is potentially ambiguous (Aissen, 2003; de Hoop and Malchukov, 2008; de Swart, 2011). In languages such as Dutch that encode who is doing what to whom by means of word order, the animacy constraint does not have grammatical effects, although it is argued to have effects on sentence processing in these languages (e.g., de Hoop and Lamers, 2006; de Swart and van Bergen, 2019). In contrast, in languages such as the Papuan language Awtuw, the animacy constraint has grammatical effects and interacts with other aspects of grammar (de Swart, 2011). In Awtuw, a noun phrase that is highest in animacy will be interpreted as the subject, unless it bears object case marking. Because of its potential to have grammatical effects, in Optimality Theory the animacy constraint is considered to be a constraint of the grammar, on a par with the word order constraint. It is this animacy constraint that has been argued to give rise to different effects in comprehension and production in child language (Hendriks et al., 2005; Hendriks, 2014, 2016).

A basic tenet of Optimality Theory is that linguistic variation – such as that between Awtuw and English, but also between child language and adult language – is characterized by a different ranking of the same constraints. Hence, language acquisition is considered a process of constraint reranking (e.g., Legendre, 2006). Several constraint reranking algorithms have been proposed to specify this process (e.g., Tesar and Smolensky, 1998; Boersma and Hayes, 2001), all showing how the linguistic input the child is exposed to leads to a (step-wise or gradual) reranking of constraints. In the adult grammar of English, the word order constraint must be stronger than the animacy constraint. This explains why English-speaking adults always select the first noun phrase as the subject in comprehension, thereby satisfying the stronger word order constraint but sometimes violating the weaker animacy constraint. If English-speaking children entertain a different ranking of the constraints and incorrectly assume the animacy constraint to be the strongest of the two constraints, the interaction between these two constraints will give rise to non-adult-like performance in comprehension, but adult-like performance in production (Hendriks et al., 2005; Hendriks, 2014, 2016). In comprehension, the stronger animacy constraint will be satisfied even if this would result in violation of the weaker word order constraint, resulting in selection of the animate noun phrase as the subject. This will yield a correct interpretation if the first noun phrase is animate, but an incorrect interpretation if the first noun phrase is inanimate. In production, in contrast, children’s immature constraint ranking will not give rise to non-adult-like performance because the animacy constraint, pertaining to meanings, is irrelevant for selecting the optimal form. As only the word order constraint is relevant in production, children, like adults, are expected to satisfy this constraint. Thus, the Optimality Theory account predicts a production-comprehension asymmetry in child language.

This study focuses on Dutch. In Dutch, like English, the dominant word order in active transitive main clauses is subject-verb-object (SVO), although main clauses allow for alternative word orders, and subordinate clauses have SOV word order. Also like English, Dutch only has overt case marking on pronouns, not on full noun phrases. According to a corpus study by Bouma (2008) using the Spoken Dutch Corpus (CGN, 2004), 70% of all Dutch main clauses begin with the subject. When a main clause begins with the direct object, which is true for 8% of main clauses if the direct object is a definite full noun phrase (Bouma, 2008), this results in object-verb-subject (OVS) word order. However, OVS word order occurs in specific discourse contexts only and requires special intonation. To explain the availability of variation between SVO and OVS word order in some contexts, as well as the lack of variation in other contexts (so-called “word order freezing”), it has been argued that constraints pertaining to information structure and definiteness are relevant as well (see Bouma and Hendriks, 2012, for an Optimality Theory account of word order in Dutch, and a corpus study testing its predictions). However, since we will only be concerned with transitive sentences in isolation with two definite arguments, these additional constraints do not play a role in the present study. In isolation, sentences like the Dutch counterpart of *The car is pushing the cow* are interpreted by Dutch adults as expressing SVO word order only, with the car doing the pushing and the cow being pushed.

Based on the theories of language acquisition just presented, we can formulate predictions about how Dutch children will comprehend and produce simple transitive sentences. In this study, children acquiring Dutch are tested on their comprehension of transitive sentences in a picture selection task and a preferential looking task, and on their production of transitive sentences in a parallel sentence elicitation task. Picture selection tasks are considered to be less demanding than act-out tasks and can be used with children starting at age 20–24 months (Gerken and Shady, 1996). Preferential looking tasks place even fewer task demands on children, since they do not require an overt response, and have been successfully used for investigating sentence comprehension with children as young as 17 months old (e.g., Hirsh-Pasek and Golinkoff, 1996; Candan et al., 2012). Based on the Optimality Theory account, we expect 2-year-old and perhaps also 3-year-old children to still make word order errors in their interpretation of transitive sentences in the picture selection task and to base their responses on the animacy of the subject and the object. In particular, they are predicted to perform best if the subject is animate and the object is inanimate. At the same time, in the sentence elicitation task, we expect these children to conform to Dutch SVO word order in their produced utterances. Alternatively, if children’s non-adult-like performance in the picture selection task is caused by task demands rather than a non-adult constraint ranking, they are expected to show better performance in the preferential looking task than in the picture selection task and to not be systematically influenced by animacy in either comprehension task.

## Experiment

### Participants

Thirty-two monolingual Dutch-speaking children participated in the study, divided into a group of fifteen 2-year-olds (age range 2;5–3;2, *M* = 2;9, 5 male) and a group of seventeen 3-year-olds (age range 3;3–4;1, *M* = 3;8, 7 male). In addition, forty-one native Dutch-speaking adults (*M* = 22 years, 12 male) served as controls. The study was carried out in accordance with the recommendations of the Research Ethics Committee CETO of the University of Groningen. The protocol was approved by CETO (review 72201140). All adult participants and parents of all child participants gave written informed consent in accordance with the Declaration of Helsinki. For each child, vocabulary scores were collected using the normed Dutch adaptation N-CDI (Zink and Lejaegere, 2003) of the MacArthur Communicative Development Inventory (MCDI; Fenson et al., 2000) for the younger age group, and KINT (Koster et al., 2004) for the older age group.

### Materials and Methods

Participants were tested on their comprehension and production of transitive sentences in four conditions, illustrated by the following sentences (sentences are in Dutch, with English translations in italics; S = subject, O = object):

(1) De koe duwt de hond. [S +animate; O +animate]
  *the cow is pushing the dog*.
(2) De hond duwt de bus. [S +animate; O –animate]
  *the dog is pushing the bus*.
(3) De auto duwt de koe. [S –animate; O +animate]
  *the car is pushing the cow*.
(4) De bus duwt de auto. [S –animate; O –animate]
  *the bus is pushing the car*.

The variables manipulated are subject animacy (+animate or –animate) and object animacy (+animate or –animate). Comprehension was tested in two tasks: a picture selection task and a preferential looking task (the latter task with children only). Production was tested in a sentence elicitation task. In addition to response accuracy in the picture selection task and produced utterances in the sentence elicitation task, gaze data was collected during all tasks for children and adults.

**FIGURE 1:**
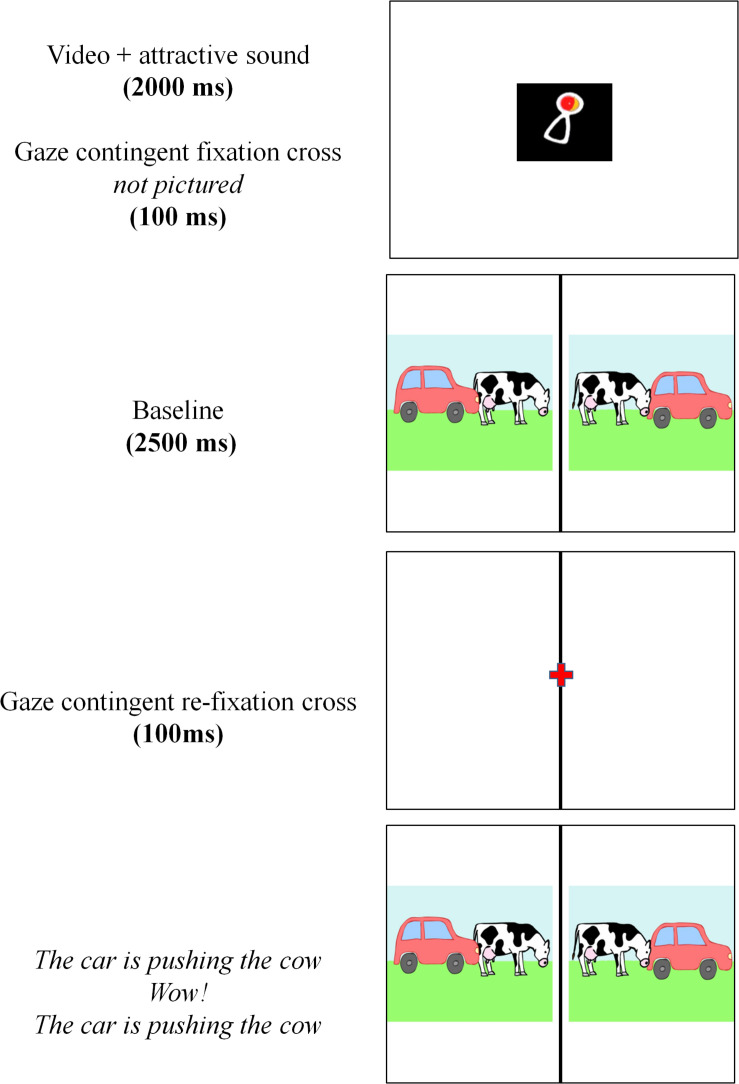
Timeline of a trial in the picture selection task and the preferential looking task.

In the two comprehension tasks, the same sentence materials were used, which are identical to those used in Cannizzaro’s (2012) act-out experiment involving animals and vehicles. The two tasks featured 4 test items per sentence type, so 16 items in total. Half of the test sentences contained the transitive verb *trekken* (‘pull’), and the other half contained the transitive verb *duwen* (‘push’). These two verbs are reversible and are felicitous with animate as well as inanimate subjects and objects. The subjects and objects were animals and vehicles familiar to young children. Each test sentence was accompanied by two colored animated pictures appearing side-by-side on a computer screen (see Figure 1). The target picture showed the action corresponding to the SO (Subject-Object) interpretation (with the first noun phrase as the subject and the second noun phrase as the object), and the distractor picture showed the same action but corresponding to the OS (Object-Subject) interpretation (with the first noun phrase as the object and the second noun phrase as the subject).

For the comprehension tasks, two versions of the sentences were created that differed in the order of the two noun phrases, to avoid effects of event probability. In addition, the items were arranged in two different orders, to avoid order effects. This resulted in a total of four lists. Each participant only saw one list. Furthermore, direction of action within the pictures and side of the target picture on the screen were balanced across conditions. In addition to the 16 test items, the comprehension tasks included 6 practice items and 4 filler items for children, and 3 practice items and 16 filler items for adults. For children, filler items were included to verify that they understood the task. For adults, filler items were included to mask the goal of the experiment.

The production task elicited the same 16 sentences that were used in the comprehension tasks. The pictures used for sentence elicitation were the target pictures of the picture pairs used in comprehension. In addition to the 16 test items, the production task included 6 practice items but no filler items for children, and 3 practice items and 16 filler items for adults.

### Procedure

The children were tested individually in the Eye Lab at the University of Groningen by two experimenters. Each child was tested in two sessions, about 1 week apart. The first session started with two pre-tests, in which naming of the animals and vehicles and of the pulling and pushing actions were practiced, without modeling word order. No child had problems naming the objects and actions. Then the preferential looking task was administered, followed by the sentence elicitation task. This order allowed us to model, through the preferential looking task, the sentence frame for the sentence elicitation task, without having to provide feedback on produced forms or repeat trials. The two tasks tested the same verb, so either *trekken* (‘pull’) or *duwen* (‘push’), to avoid suboptimal performance due to confusion between the two verbs. Next, the picture selection task was administered with the other verb. The second session followed the same procedure with the remaining test items and had the same order of tasks. Gaze data in all three tasks was collected using a Tobii T120 remote eye-tracker at a frame rate of 60 Hz.

In the picture selection task, the child heard a sentence and was instructed to point to the picture that matched the sentence. Figure 1 shows the timeline of a trial in the picture selection task. Each trial was preceded by an attention-getting image and sound for 2000 ms. Subsequently, a gaze contingent fixation cross appeared in the center of the screen. Once the child had fixated on the cross for 100 ms, the two animated pictures appeared on the screen without auditory input. This baseline of 2500 ms was followed by a second fixation cross. This cross had to be fixated on by the child for 100 ms before the pictures were displayed again, this time with the prerecorded test sentence, an exclamation (e.g., “Look!”), and a repetition of the test sentence. The pictures remained visible until the child made a decision by a pointing gesture. The pointing gestures were scored by both experimenters.

In the preferential looking task, the child heard a sentence and merely had to watch the animated pictures, without having to give a response. Apart from this, the timeline of a trial in the preferential looking task was the same as in the picture selection task. The pictures remained visible on the screen for 7000 ms.

In the sentence elicitation task, the child heard no audio and saw only a single animated picture on the full screen. The child was instructed to tell a hand puppet who had closed his eyes what was happening in the picture. All elicited sentences were audio recorded. In addition, we measured the participant’s voice onset latency (VOL), which is the time between the presentation of a visual stimulus and the beginning of the speaker’s sentence (Bock et al., 2004). VOLs were used to synchronize the collected gaze data to the onset of the elicited sentence.

The procedures described above were specifically tailored to optimally test young children. The procedures used for adults were adapted accordingly, since the main reason for testing adults was to establish the target pattern of production and comprehension of simple transitive constructions in isolation, given the variation in word order in Dutch main clauses. Adult participants were tested on comprehension and production in one session. They did not receive a preferential looking task, because the task was believed to be too simple and boring for adults, potentially giving rise to task-unrelated looking behavior. The order of the two tasks was balanced, with half the adults receiving the picture selection task first and the other half receiving the sentence elicitation task first. In the sentence elicitation task, adults were instructed to describe the animated picture in a short sentence (with no hand puppet necessary). In the picture selection task, adults were instructed to press one of two marked keys to indicate which of the two pictures matched the sentence they heard. In addition to their responses and gaze data, their reaction times (RTs) were recorded.

### Scoring

The participants’ responses in the picture selection task were categorized as *SO interpretation*, *OS interpretation*, or *Unscorable*. A response was categorized as SO interpretation if the participant chose the target picture reflecting the SO interpretation. A response was categorized as OS interpretation if the participant chose the distractor picture reflecting the OS interpretation. Unscorable responses included items for which the child pointed to both pictures, did not give any response, or did not give a clear response; items not administered due to a technical error were also categorized as unscorable. Only SO interpretations and OS interpretations were included in the analysis.

The produced utterances in the sentence elicitation task were first transcribed by the first author. The utterances of 10% of the participants were transcribed by a second transcriber, resulting in 91% agreement on the adult utterances and 90% agreement on the child utterances. Next, the first author and a second scorer independently categorized all utterances. If there was a disagreement between the two scorers, a third scorer made a final decision. Produced utterances were categorized as *SO word order* (with the subject preceding the object), *OS word order* (with the object preceding the subject), or *Unscorable*. Scorable utterances did not have to be complete utterances but, when incomplete, did require a finite verb to allow us to distinguish the SVO word order of Dutch main clauses from the SOV word order of Dutch non-finite clauses. If a participant produced SVO word order, or SV or VO word order with a finite verb, this was categorized as SO word order. Utterances with OVS word order, or OV or VS word order with a finite verb, were categorized as OS word order. Unscorable utterances included insufficient or unclear responses in which word order could not be determined, missing responses, and responses that did not contain the target verb or a synonym. Utterances with a non-finite verb and only one noun or passives were also categorized as Unscorable. Inter-scorer agreement in categorizing the produced utterances was high (adults: Cohen’s κ = 0.94; children: Cohen’s κ = 0.90).

## Results

In this section we present the results of the study (see also Cannizzaro, 2012), starting with adults’ and children’s response accuracy and adults’ RTs in the picture selection task. This is followed by adults’ and children’s produced utterances in the sentence elicitation task. We then present the gaze patterns in the picture selection task (for adults) and the preferential looking task (for children), and the gaze patterns for both groups in the sentence elicitation task. Finally, we compare the scorable responses in the picture selection and sentence elicitation tasks.

The results were analyzed using linear mixed-effects modeling (e.g., Baayen, 2008; Baayen et al., 2008). We compared different models using a simplification procedure to determine the model with the best fit. This procedure starts with creating a maximal model including all possible three-way and two-way interactions and main effects. This maximal model is then compared to a simpler model without the three-way interaction using a chi-square test that evaluates each model’s goodness of fit (Baayen, 2008; Matuschek et al., 2017). If a simpler model has a significantly lower goodness of fit than the more complex model, removal of the interaction or factor is not justified. This model comparison procedure is repeated until the model with the best fit has been determined. The analyses were carried out using the software package R (R Core Team, 2020), version 2.13. The *lmer* function in the package lme4 was used to obtain coefficient estimates for all data, and additionally *p*-values for binary data; *z*-statistic is reported (Bates, 2007). The *pvals.fnc* function in package languageR was used to obtain *p*-values for continuous data using Markov Chain Monte Carlo sampling; *t*-statistic is reported (Baayen, 2008; Baayen et al., 2008).

### Results of the Picture Selection Task

Participants were excluded from the analysis of the picture selection task if they did not contribute at least two scorable responses per sentence type. For this reason, two of the fifteen 2-year-olds were excluded. The comprehension results of the adults are presented in Table 1, and of the children in Table 2.

**TABLE 1:** Adults’ mean proportions of SO interpretations (and standard deviations) in the picture selection task (Comprehension) and their mean proportions of SO word order (and standard deviations) in the sentence elicitation task (Production), per animacy condition.

Sentence Type	Comprehension	Production
	**Adults (*n* = 41)**	**Adults (*n* = 38)**
[S +animate; O +animate]	0.98 (0.08)	1.00 (−)
[S +animate; O –animate]	0.99 (0.04)	1.00 (−)
[S –animate; O +animate]	0.94 (0.12)	1.00 (−)
[S –animate; O –animate]	0.96 (0.09)	1.00 (−)
Total	0.97 (0.04)	1.00 (−)

**TABLE 2:** Children’s mean proportions of SO interpretations (and standard deviations) in the picture selection task (Comprehension) and their mean proportions of SO word order (and standard deviations) in the sentence elicitation task (Production), per animacy condition and age group.

Sentence type	Comprehension		Production	
	2-year-olds (*n* = 13)	3-year-olds (*n* = 17)	2-year-olds (*n* = 5)	3-year-olds (*n* = 16)
[S +animate; O +animate]	0.40 (0.25)	0.73 (0.23)	1.00 (−)	0.93 (0.18)
[S +animate; O –animate]	0.72 (0.26)	0.79 (0.25)	0.90 (0.22)	0.96 (0.17)
[S –animate; O +animate]	0.48 (0.27)	0.54 (0.28)	0.60 (0.25)	0.92 (0.18)
[S –animate; O –animate]	0.54 (0.29)	0.71 (0.27)	0.73 (0.28)	0.88 (0.21)
Total	0.54 (0.10)	0.70 (0.18)	0.81 (0.13)	0.92 (0.17)

Of the responses in the picture selection task, 655 of the 656 responses of the adults were scorable (only one response was unscorable due to a technical error), and 195 of the 208 responses of the 2-year-olds and 266 of the 272 responses of the 3-year-olds were scorable.

To determine whether animacy affected response accuracy in adults’ comprehension, the binomial data (SO vs. OS interpretation) was fit to a linear mixed-effects model with subject animacy (animate vs. inanimate) and object animacy (animate vs. inanimate) as fixed factors, and participants and items as random factors. Since there was no significant two-way interaction [χ^2^(1) = 0.46, *p* > 0.1], we checked for main effects in the baseline model. There was no main effect of object animacy [χ^2^(1) = 2.19, *p* > 0.1], but there was a main effect of subject animacy [χ^2^(1) = 4.65, *p* = 0.03], with lower response accuracy on sentences with an inanimate subject (β = −0.61; *z* = −2.08; *p* = 0.04). The inclusion of the control factors test verb (*push* vs. *pull*), first task (comprehension first vs. production first), target side, and list did not significantly explain more variance in the data. Thus, Dutch adults gave SO interpretations to the sentences they heard 97% of the time, and were more likely to do so when the subject was animate.

To determine whether animacy affected response accuracy in children’s comprehension, the binomial data (SO vs. OS interpretation) was fit to a model with subject animacy, object animacy, and age group as fixed factors, and participants and items as random factors. There were no significant three-way [χ^2^(1) = 2.37, *p* > 0.1] or two-way [χ^2^(3) = 2.68, *p* > 0.1] interactions between the fixed predictors. Since including interactions was not justified, we checked the baseline model for main effects. There were three distinct main effects that were significant predictors of response accuracy. There was a main effect of age group [χ^2^(1) = 8.20, *p* = 0.004], with the older children more likely to choose SO interpretations than the younger children (β = 0.37; *z* = 3.06; *p* = 0.002); a main effect of subject animacy [χ^2^(1) = 5.67, *p* = 0.02], with all children more likely to choose SO interpretations when the subject was animate (β = 0.24; *z* = 2.39; *p* = 0.02); and a main effect of object animacy [χ^2^(1) = 12.58, *p* < 0.001], with all children more likely to choose SO interpretations when the object was inanimate (β = −0.36; *z* = −3.54; *p* < 0.001). The inclusion of the control factors gender, test verb, target side, list and vocabulary score did not significantly explain more variance in the data.

Thus, 3-year-olds were more likely to choose SO interpretations (70%) than 2-year-olds (54%), children were more likely to choose SO interpretations when the subject was animate, and children were more likely to choose SO interpretations when the object was inanimate.

In addition to responses, for the adults we also collected RTs. Items with OS interpretations (*n* = 21) or extreme RTs (*n* = 2) were removed from the RT analysis. Extreme RTs were considered those outside 3 standard deviations of the participant’s personal mean. Mean RTs on the four sentence types are shown in Table 3.

**TABLE 3:** Adults’ mean reaction times in ms (and standard deviations) for giving SO interpretations in the picture selection task (Comprehension), per animacy condition.

Sentence type	Reaction times (sd)
	**Adults (*n* = 41)**
[S +animate; O +animate]	2001 (392)
[S +animate; O –animate]	1973 (391)
[S –animate; O +animate]	2301 (526)
[S –animate; O –animate]	2294 (591)
Total	2140 (451)

To determine whether animacy affected RTs in the picture selection task, the log transformed RTs were fit to a model with subject and object animacy as fixed factors, and participants and items as random factors. Since including an interaction was not justified [χ^2^(1) = 0, *p* = 1], we checked the baseline model for main effects. There was no main effect of object animacy [χ2(1) = 0.10, *p* > 0.1], but there was a main effect of subject animacy [χ^2^(1) = 15.68, *p* < 0.001]. The adults had longer RTs when the subject was inanimate (β = 0.07; *t* = 4.35; *p* < 0.001). The inclusion of the control factors test verb, first task, target side, and list did not significantly explain more variance in the data.

Thus, adults were faster to select the SO interpretation when the subject was animate.

### Results of the Sentence Elicitation Task

Participants were excluded from the analysis of the sentence elicitation task if they did not contribute at least two scorable responses per sentence type. For this reason, three of the 41 adults were excluded due to too many utterances that did not contain the target verb or a synonym. Furthermore, ten of the fifteen 2-year-olds were excluded (including the two who were also excluded from the picture selection analysis), and one of the seventeen 3-year-olds. In Tables 1, 2, the production results of the remaining adults and children are presented.

In the sentence elicitation task, 577 of the 608 responses of the adults were scorable (29 were unscorable because of the use of a non-target verb, and 2 because of the use of a passive), and 70 of the 80 responses of the 2-year-olds and 232 of the 256 responses of the 3-year-olds were scorable.

Because the adults used SO order 100% of the time, their production data was not further analyzed.

To determine whether animacy affected response accuracy in children’s production, the binomial data (SO vs. OS order) was fit to a model with subject animacy, object animacy, and age group as fixed factors, and participants and items as random factors. The maximal model was not fit due to complete collinearity between the three-way interaction of the fixed predictors and the two-way interaction between age group and subject animacy. The three-way interaction was therefore not included in the model, as a strategy to reduce this severe collinearity (Baayen, 2008, p. 183). There were no significant two-way interactions [χ^2^(3) = 1.93, *p* > 0.1]. In the baseline model, there was no main effect of object animacy [χ^2^(1) = 1.94, *p* > 0.1]. Subject animacy was a significant predictor [χ^2^(1) = 8.87, *p* = 0.003], with the children more likely to use SO order when the subject was animate (β = 0.81; *z* = 2.97; *p* = 0.003). The inclusion of the control factors gender, test verb, direction of action, list and vocabulary score showed that both test verb [χ^2^(1) = 12.20, *p* < 0.001] and direction of action [χ^2^(1) = 4.63, *p* = 0.03] significantly explain more variance in the data, with the children more likely to use SO order when the verb was *push* (β = 0.90; *z* = 3.18; *p* = 0.001) as well as when the direction of the action was to the left (β = 0.54; *z* = 2.05; *p* = 0.04).

So in general children were more likely to produce SO word order when the subject was animate.

### Gaze Patterns in Sentence Comprehension

Because the picture selection task required an overt response (pointing) that could have been demanding for the children, we also investigated their gaze patterns in a task not requiring an overt response, namely a preferential looking task. We compare children’s gaze patterns in this task to adults’ gaze patterns collected in the picture selection task.

For adults, items that had track loss of both eyes for more than 33% of the trial from presentation to button press were removed from the gaze analysis. For children, we used a less conservative threshold than for adults and removed items that had track loss of both eyes for more than 33% of the four-second time interval after presentation of the auditory stimulus. Even with this less conservative threshold, children’s gaze data in the picture selection task could not be used due to too much track loss because they moved around a lot when pointing. Therefore, we only analyzed children’s gaze data gathered during the preferential looking task. These data are compared to the adults’ gaze data gathered during the picture selection task.

All adult and child participants were included in the gaze analyses: the adults in the gaze analyses of the picture selection task and the children in the gaze analyses of the preferential looking task. Before analysis, we removed test items of adults with too much track loss (*n* = 3), OS interpretations (*n* = 21), and extreme RTs (*n* = 2) from the gaze analysis of the picture selection task. Due to too much track loss, we removed 70 test items of children from the gaze analysis of the preferential looking task.

Areas of interest (AOIs) in the pictures were labeled as *Target picture*, *Distractor picture*, and *Not on AOI*. To determine the effects of animacy on sentence processing, we looked at the participants’ eye gaze for each of the four sentence types in four time windows: Time window 1 runs from the start of the trial to the offset of the sentence subject and has a duration of about 600 ms, and time windows 2, 3, and 4 are subsequent intervals of 1000 ms following the offset of the sentence subject.

**FIGURE 2:**
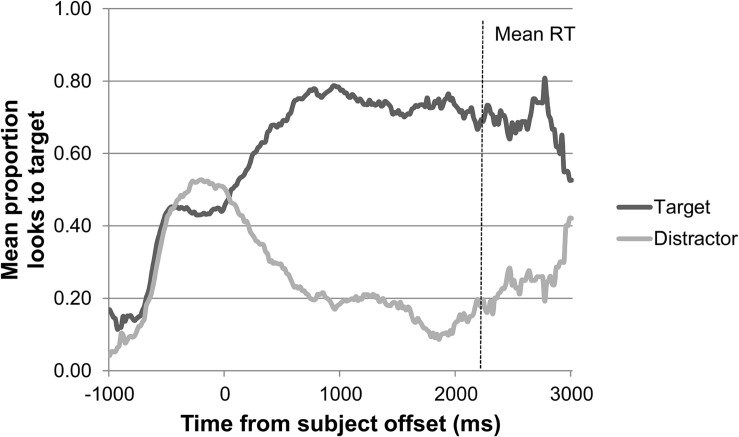
Adults’ pattern of looks to target versus distractor over the course of a trial in the picture selection task (*n* = 41).

The general adult pattern of looks to target and distractor in the picture selection task over the course of a trial, synchronized to the offset of the sentence subject, is shown in Figure 2. The adults show a pattern of looking at the target within the first 1000 ms following the offset of the subject.

**FIGURE 3:**
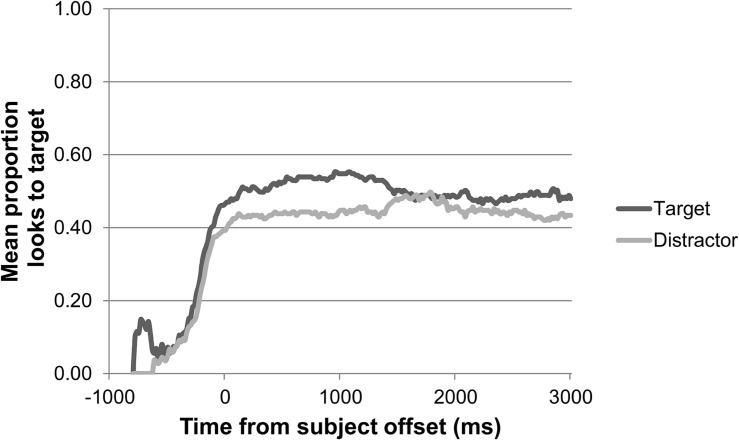
2-year-olds’ pattern of looks to target versus distractor over the course of a trial in the preferential looking task (*n* = 15).

**FIGURE 4:**
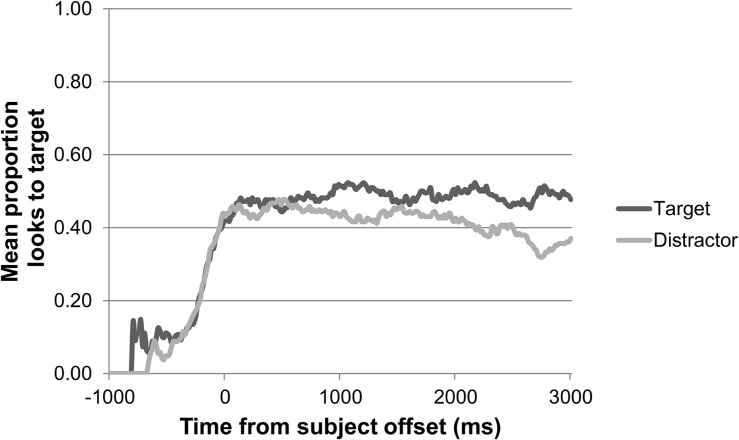
3-year-olds’ pattern of looks to target versus distractor over the course of a trial in the preferential looking task (*n* = 17).

For children, we first inspected their eye gaze during the 2500 ms baseline for any initial preference for either target or distractor picture. Neither age group showed an initial preference for target or distractor picture in any of the four animacy conditions during the baseline. The children’s general pattern of looks to target and distractor during the preferential looking task over the course of a trial, synchronized to the offset of the subject, is shown in Figure 3 for the 2-year-olds, and in Figure 4 for the 3-year-olds. These gaze plots show that, in general, children’s mean proportions of looks to the target did not reach above 0.60 during the 3000 ms following the offset of the subject.

**FIGURE 5:**
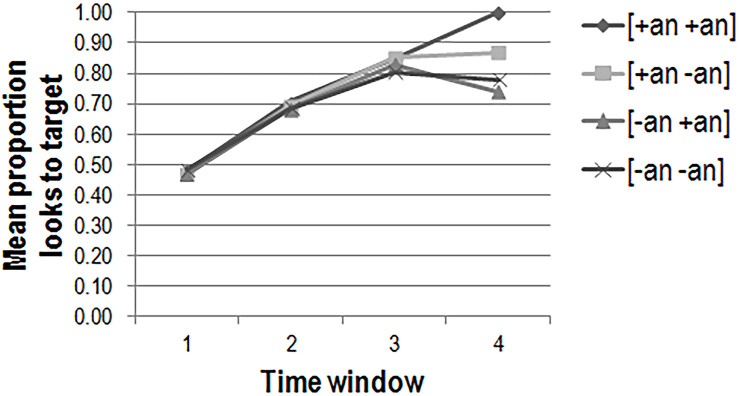
Adults’ mean proportions of looks to target per condition in each of the four time windows in the picture selection task (*n* = 41).

**FIGURE 6:**
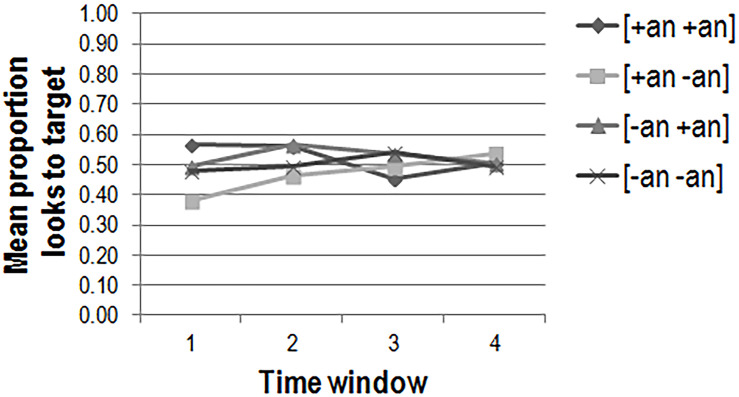
2-year-old’s mean proportions of looks to target per condition in each of the four time windows in the preferential looking task (*n* = 15).

**FIGURE 7:**
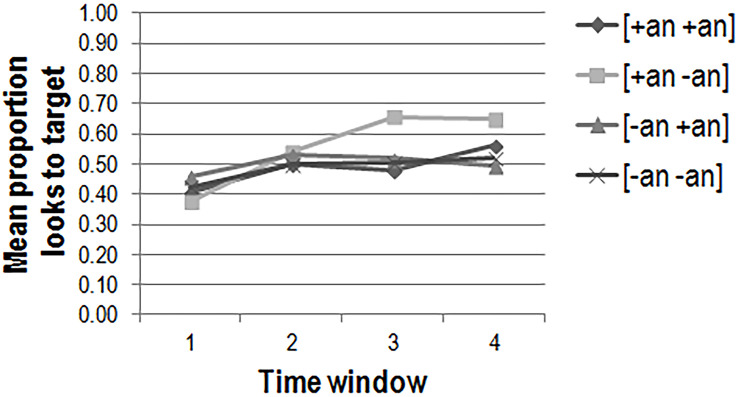
3-year-old’s mean proportions of looks to target per condition in each of the four time windows in the preferential looking task (*n* = 17).

The mean proportions of looks to target per animacy condition in each of the four time windows are plotted in Figure 5 (adults), Figure 6 (2-year-olds), and Figure 7 (3-year-olds).

To investigate the effects of animacy on sentence processing, we first consider adults’ gaze. To determine whether animacy affected which AOI was fixated on by the adults during picture selection, the empirical logit transformed (Agresti, 2002, p. 87; Jaeger, 2008, p. 442) mean looks to target were fit to a model with subject animacy, object animacy, and time window as fixed factors, and participant and item as random factors. There was no significant three-way interaction between the fixed predictors [χ^2^(1) = 0.18, *p* > 0.1]. There was a significant interaction of time window and subject animacy [χ^2^(1) = 11.90, *p* < 0.001] as well as a significant main effect of time window [χ^2^(1) = 711.94, *p* < 0.001]. Thus, the adults looked increasingly toward the target picture as time progressed (β = 1.98; *t* = 30.09; *p* < 0.001), but did so to a significantly lesser degree when the subject was inanimate (β = −0.22; *t* = 3.45; *p* < 0.001). The inclusion of the control factors test verb, first task, target side, and list showed that target side significantly explained more variance in the data [χ^2^(1) = 38.66, *p* < 0.001], with participants more likely to fixate on the target picture if it was on the left (β = 0.48; *t* = 8.04; *p* < 0.001). The inclusion of an interaction of target side and time window was also a significant improvement [χ^2^(1) = 101.71, *p* < 0.001], indicating that the effect of target side decreased as time progressed (β = −0.64; *t* = 10.24; *p* < 0.001).

We carried out the same analysis for the gaze data collected for 2-year-old children in the preferential looking task. There were no three-way [χ^2^(1) = 0.07, *p* > 0.1] or two-way [χ^2^(1) = 1.10, *p* > 0.1] interactions between the fixed predictors. In the baseline model there was only a significant effect of time window [χ^2^(1) = 19.58, *p* < 0.001], indicating that the 2-year-olds looked increasingly toward the target picture as time progressed in general (β = 0.34; *t* = 4.45; *p* < 0.001). The inclusion of the control factors gender, test verb, target side, and list showed that target side significantly explains more variance in the data [χ^2^(1) = 21.36, *p* < 0.001], with children more likely to fixate on the target picture if it was on the left (β = 0.68; *t* = 5.05; *p* < 0.001). Target side appeared not to interact with time window [χ^2^(1) = 0.10; *p* > 0.10], as it had for adults.

The same analysis was also carried out for the 3-year-olds. There was no three-way [χ^2^(1) = 0.97, *p* > 0.1] interaction between the fixed predictors. There was a two-way interaction between time window and subject animacy [χ^2^(1) = 4.39, *p* = 0.04]. Together with the main effect of time window [χ^2^(1) = 27.61, *p* < 0.001], this indicates that the 3-year-olds looked increasingly toward the target picture as time progressed in general (β = 0.36; *t* = 5.29; *p* < 0.001) and that this effect was intensified when the subject was animate (β = 0.14; *t* = 2.10; *p* = 0.04). The inclusion of the control factors gender, test verb, target side, and list did not significantly explain more variance in the data.

Summarizing, as the sentence unfolds, adults look more toward the target picture reflecting the SO interpretation, but this effect is less strong when the subject is inanimate. Children also look more toward the target picture as the sentence unfolds. However, while this effect is intensified in 3-year-olds when the subject of the sentence is animate, no effect of animacy is found in 2-year-olds.

### Gaze Patterns in Sentence Production

Gaze data was also collected from adults and children during the sentence elicitation task. Only those adults and children who remained in the accuracy analyses of the sentence elicitation task were included in the gaze analyses of this task. One additional adult was excluded from the gaze data analysis due to extreme track loss. Of the data from the remaining 37 adults, test items with extreme track loss (*n* = 8) or extreme VOLs (*n* = 5) were removed. Extreme VOLs were considered those outside 3 standard deviations of the participant’s personal mean. Furthermore, one 3-year-old child was excluded from the gaze analysis of the production task because he did not have at least two validly tracked items on at least two sentence types. Of the data from the remaining 20 children (15 fifteen 3-year-olds and five 2-year-olds), test items with extreme track loss (*n* = 56) as well as incorrect OS utterances (*n* = 30) were removed from the analysis.

Within each picture, AOIs were labeled as *Agent*, *Patient* and *Not on AOI*. Analysis was done over two time windows: Time window 1 is the interval of 1000 ms prior to the onset of the subject, and time window 2 is the interval of 1000 ms after the onset of the subject. For each time window, we calculated the difference between the proportion of looks to the agent and the proportion of looks to the patient (the so-called “agent advantage score”).

**FIGURE 8:**
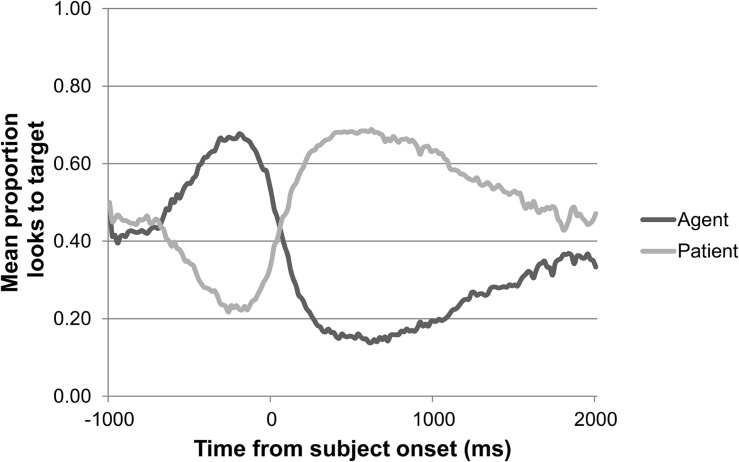
Adults’ pattern of looks to agent versus patient over the course of a trial in the sentence elicitation task (*n* = 37).

Figure 8 shows a gaze plot of the general adult pattern of looks to agent and patient over the course of a trial, synchronized to the onset of each participant’s sentence. The gaze plot shows that the adults looked first to the agent, expressed as the subject of the sentence, prior to starting a sentence, and then to the patient, expressed as the object of the sentence.

**FIGURE 9:**
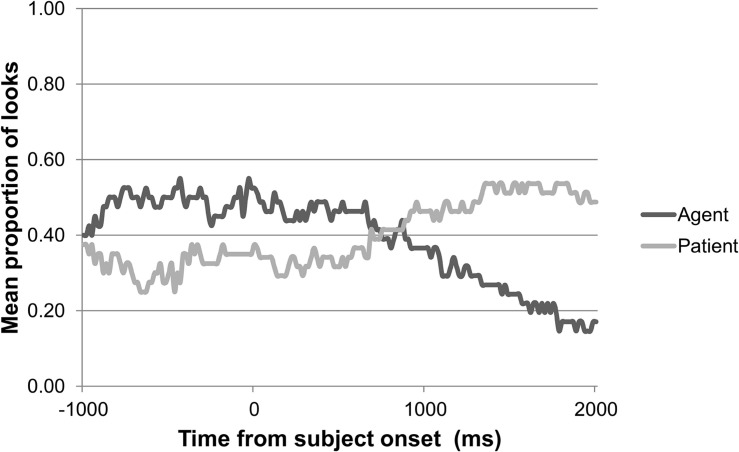
2-year-olds’ pattern of looks to agent versus patient over the course of a trial in the sentence elicitation task (*n* = 5).

**FIGURE 10:**
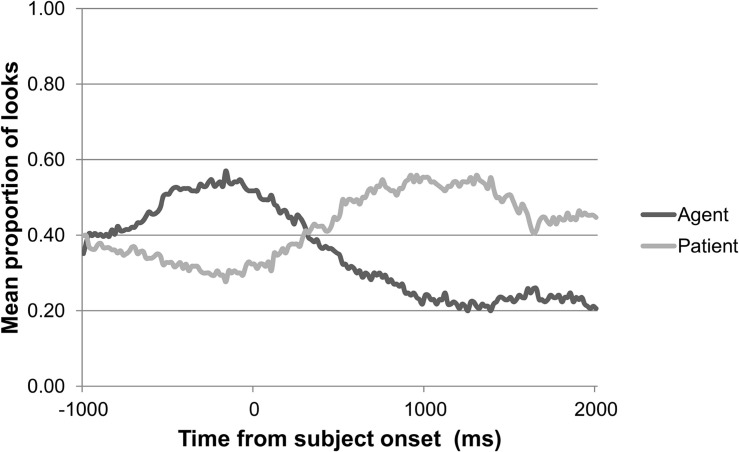
3-year-olds’ pattern of looks to agent versus patient over the course of a trial in the sentence elicitation task (*n* = 15).

As Figures 9, 10 show, the 2-year-olds and 3-year-olds also looked first to the agent and then to the patient while producing the sentence. The 2-year-olds took about 750 ms and the 3-year-olds took about 250 ms after starting their sentence to shift their gaze from agent to patient. Thus, the eye gaze of the Dutch-speaking adults as well as the children reflect a search for agent followed by a search for patient.

To determine whether animacy affected which AOI was fixated on by adults during sentence planning and production, the empirical logit transformed mean agent advantage scores from each time window were fit to a model with subject animacy, object animacy, and time window as fixed factors, and participant and item as random factors. There was a significant three-way interaction between the fixed predictors [χ^2^(1) = 5.89, *p* = 0.02], which could be interpreted in light of a significant effect of time window [χ^2^(1) = 260.69, *p* < 0.001]. In all sentence types, there was a decrease in the preference for agent over patient from the first to the second time window (β = −4.16.; *t* = 17.18; *p* < 0.001), but to a significantly lesser degree in the sentences with an animate subject and an inanimate object (β = −0.58; *t* = −2.43; *p* < 0.02). Thus, the adults looked more to the patient as the sentence unfolded, but less so for sentences with an animate subject and an inanimate object. The inclusion of the control factors test verb, first task, direction of action, and list showed that the inclusion of the two-way interaction between verb and time window significantly explained more variance in the data [χ^2^(1) = 94.44, *p* < 0.001], with greater looks to the agent over patient in the first time window when the verb was *pull* (β = 4.26.; *t* = 9.01; *p* < 0.001).

To determine whether animacy affected which AOI was fixated on by the 2- and 3-year-olds during sentence planning and production, the empirical logit transformed mean agent advantage scores from each time window were fit to a model with subject animacy, object animacy, time window, and age group as fixed factors, and participant and item as random factors. There were no significant four-way [χ^2^(1) = 0.07, *p* > 0.1], three-way [χ^2^(4) = 1.30, *p* > 0.1], or two-way [χ^2^(6) = 5.57, *p* > 0.1] interactions between the fixed predictors. In the baseline model there was no effect of subject animacy [χ^2^(1) = 1.10; *p* > 0.1] or object animacy [χ^2^(1) = 0.31; *p* > 0.1]. There was a significant main effect of time window [χ^2^(1) = 16.51; *p* < 0.001], with the agent advantage score decreasing from the first to the second time window (β = −1.68; *t* = −4.09; *p* < 0.001).

As we should be cautious in our interpretation of the 2-year-olds’ gaze data in production because of the considerable data loss, we ran a second analysis with only the 3-year-old children. Again, there were no significant three-way [χ^2^(1) = 0.03; *p* > 0.1], or two-way [χ^2^(3) = 3.93; *p* > 0.1] interactions between the fixed predictors. In the baseline model there was no effect of subject animacy or object animacy, but there was a significant main effect of time window [χ^2^(1) = 503.21; *p* < 0.001], with the agent advantage score decreasing from the first to the second time window (β = −1.95; *t* = −4.37; *p* < 0.001). Thus, the results of the 3-year-olds only are similar to the results of the children including the five 2-year-olds.

The inclusion of the control factors gender, test verb, direction of action, and list showed that the test verb significantly explained more variance in the data. For both the model with the 2-year-olds [χ2(1) = 12.17; *p* < 0.001] and the model without the 2-year-olds [χ2(1) = 12.88; *p* < 0.001], there were greater looks to the agent when the verb was *pull* (β = 1.11; *t* = 3.51; *p* < 0.001 and β = 1.05; *t* = 3.67; *p* < 0.001, respectively).

Summarizing, adult speakers look less to the agent and more to the patient when producing the subject of the sentence, but this effect is less strong when the subject is animate and the object is inanimate. No effects of animacy are found for children’s eye gaze during sentence production.

### Comparing Comprehension and Production

To determine whether there was a difference between children’s use of word order in comprehension and production, in a separate analysis we compare children’s SO interpretations in comprehension with their produced SO word order in production. However, since scorability appeared to be higher in the picture selection task than in the sentence elicitation task, we first need to rule out the possibility that scorability influenced our results, since variation in children’s ability to produce scorable responses could be due to animacy. Therefore, we need to establish whether there was an effect of animacy condition on scorability in comprehension or production.

Mean scorability in the picture selection task, based on the children who had been included in the analysis of this task, was high and ranged between 92 and 100% per animacy condition. To determine whether animacy affected scorability on this task, the binomial data (scorable vs. unscorable) were fit to a linear mixed-effects model. Subject animacy, object animacy, and age group were included as fixed factors, and participants and items as random factors. There were no significant three-way or two-way interactions between the fixed predictors, so only baseline results were inspected for the factors age group, subject animacy, and object animacy. Results showed that neither age group [χ2(1) = 3.53, *p* = 0.06] nor subject [χ2(1) = 1.23, *p* > 0.1] or object animacy [χ2(1) = 0, *p* > 0.1] had a significant influence on scorability in the picture selection task.

Mean scorability in the sentence elicitation task, based on the children who had been included in the analysis of this task, ranged between 80 and 95% per animacy condition. The same analysis was run as described for the picture selection task. There were no significant three-way or two-way interactions between the fixed predictors. Overall, there was no effect of age group [χ2(1) = 1.53, *p* > 0.1] nor of subject [χ2(1) = 0.17, *p* > 0.1] or object animacy [χ2(1) = 1.41, *p* > 0.1] on scorability in production.

Thus, in production as well as comprehension, the unscorable items were distributed evenly across animacy conditions. We interpret this as justification that the results from the picture selection task and the sentence elicitation task can be compared, although the tasks may place different demands on the children. The analysis that follows is based on the items for which in both comprehension and production the child gave a scorable response. The SO and OS responses (i.e., selected interpretations and produced word orders) for these items per age group are shown in Table 4.

**TABLE 4:** Children’s responses in numbers (and percentages) of items for which they gave a scorable response for both comprehension and production, as SO versus OS interpretations in the picture selection task (Comprehension) and SO versus OS word order the sentence elicitation task (Production), per age group.

Response	Comprehension		Production	
	2-year-olds	3-year-olds	2-year-olds	3-year-olds
SO	55 (61.8%)	166 (70.9%)	72 (80.9%)	217 (92.7%)
OS	34 (38.2%)	68 (29.1%)	17 (19.1%)	17 (7.3%)

In order to determine whether there was a difference in performance between the sentence elicitation task and the picture selection task on the basis of these items, the binomial data (SO vs. OS) was fit to a model with task and age group as fixed factors, and participant and item as random factors. There was no significant interaction of task and age group [χ^2^(1) = 2.40, *p* > 0.1]. In the baseline model, there was a significant effect of task [χ^2^(1) = 48.20, *p* < 0.001], with children more likely to give SO responses in production than comprehension (β = 0.74; *z* = 6.57; *p* < 0.001). There was also a significant effect of age group [χ^2^(1) = 4.95, *p* = 0.03], with older children more likely to give SO responses than younger children (β = 0.34; *z* = 2.33; *p* = 0.02).

In sum, the older children were more likely to give SO responses than the younger children, and all children were more likely to give SO responses in production than in comprehension.

## Discussion

This study investigated how 2- and 3-year-old Dutch-speaking children use word order in their comprehension and their production of transitive constructions, and to what degree their use of word order is influenced by the animacy of the grammatical subject and object. The children in this study did not yet show adult-like comprehension of transitive sentences in the picture selection task: the 2-year-olds performed more poorly than the 3-year-olds and selected the correct subject-object interpretation in only 54% of cases, while the 3-year-olds did so in 70% of cases. At the same time, both age groups seem to show more advanced performance on their production of transitive sentences in the sentence elicitation task: the 2-year-olds produced subject-object word order in 81% of cases, and the 3-year-olds even did so in 92% of cases.

Comparing children’s performance on items for which they gave a scorable response in both comprehension and production, they were found to give more accurate responses corresponding to SVO word order in production than in comprehension. These results are mainly based on the 3-year-olds, since many of the 2-year-olds did not produce a sufficient number of scorable responses in production to be included in this comparison. This suggests that this asymmetry between production and comprehension is a pattern that is still firmly present in Dutch-speaking 3-year-olds. Note that the asymmetry observed in this study is not caused by differences in scorability between the conditions in the two tasks, nor by differences in children’s vocabulary development, as none of our analyses showed an effect of vocabulary score. The results of the picture selection task and the parallel sentence elicitation task thus confirm the results of earlier studies using an act-out methodology (Cannizzaro, 2012, for Dutch; Chapman and Miller, 1975; McClellan et al., 1986, for English) that also observed an asymmetry between children’s production and their comprehension of word order.

If this asymmetry between production and comprehension were an artifact of picture selection tasks and act-out tasks caused by their cognitive demands, we would expect children’s eye gaze in the minimally demanding preferential looking task to show an adult-like pattern. The adults looked more toward the target picture corresponding to the correct interpretation within the first 1000 ms following the offset of the subject, with a mean proportion of looks to this picture of almost 0.80. Like the adults in the picture selection task, the children in the preferential looking task also looked more toward the target picture as the sentence unfolded. This indicates that the children possess some knowledge of the SVO word order of Dutch main clauses. Nevertheless, the children’s mean proportions of looks to the target picture did not exceed 0.60 during the entire 3000 ms time window that was analyzed, suggesting only a weak preference for subject-object word order in comprehension. However, it cannot be ruled out that the different gaze patterns of adults and children are an effect of the different comprehension tasks used: the adults’ gaze data was collected in a picture selection task, while the children’s gaze data was collected in a preferential looking task. Although the preferential looking task was included because its task demands are believed to be low, it may have given rise to task-unrelated looking behavior in the older children, thus explaining their deviant gaze pattern compared to adults’. Indeed, according to Ambridge and Lieven (2011, p. 234–235) preferential looking tasks are seldom used with 3-year-olds because children this age find the task too easy and hence fail to pay attention. But note that the 3-year-olds in our study still did not show ceiling performance in the picture selection task, which does not suffer from this shortcoming. So although the 3-year-olds in our study may have found the preferential looking task too easy, the linguistic aspects of the task are still challenging for them.

In the picture selection task as well as the preferential looking task, children saw two animated pictures side-by-side on a computer screen, while they only saw one animated picture in the sentence elicitation task. However, it is unlikely that the simultaneous presentation of two animated pictures made these comprehension tasks too demanding for the children in our study, as several studies have successfully used intermodal preferential looking tasks with children well below age 2 (e.g., Hirsh-Pasek and Golinkoff, 1996; Candan et al., 2012). In fact, the first successful application of this task was with 4-month-old infants, who saw two events while hearing a non-linguistic auditive stimulus matching one of the events (Spelke, 1979). Thus, the comprehension tasks do not seem to be more difficult for children than the sentence elicitation task, which is supported by the higher scorability of children’s responses in comprehension compared to their responses in production.

The observed asymmetry between production and comprehension cannot be explained by an overestimation of children’s knowledge of word order in production (cf. Bates et al., 1995) either. First, the design of the sentence elicitation task was such that a correct response could not be given by merely repeating sentences that were heard before. Second, the animated pictures did not provide any clues for word order, only for agenthood. And third, in the sentence elicitation task the gaze patterns of the children who correctly produced utterances with subject-object word order were similar to the adults’ gaze patterns, but merely delayed in time. Both children’s and adults’ gaze patterns reflected a search for the agent followed by a search for the patient, as was also found by Griffin and Bock (2000) for adult speakers of English. This suggests that the underlying processes of production in adults and children are the same.

Taken together, the two comprehension tasks and the production task thus reveal an asymmetry between production and comprehension in children’s acquisition of transitive constructions in Dutch that does not seem to be explained by task effects. The finding of more advanced production than comprehension is in line with the predictions of the constraint-based Optimality Theory account of children’s acquisition of word order in transitive constructions (Hendriks et al., 2005; Hendriks, 2014, 2016). In contrast, this asymmetry is not predicted by generative and constructivist approaches and may be challenging for them to explain. The observation of this production-comprehension asymmetry in children’s acquisition of transitive constructions suggests that the form and the meaning of a transitive construction (for example, the form and the meaning of the transitive frame for pushing) are not acquired together. Instead, the form of the transitive construction seems to be acquired partly independently of its meaning. This follows from an Optimality Theory account, where production proceeds partly independently from comprehension (Smolensky, 1996; Hendriks, 2014, 2016). In this account, the pairing of form with meaning that characterizes linguistic constructions (e.g., Goldberg, 2006) gradually emerges as the by-product of acquiring the constraint ranking of the language. Only when the mapping from an input meaning to the optimal form in production and the mapping from an input form to the optimal meaning in comprehension result in the same form-meaning pairing, as happens under the adult ranking of the constraint but not yet young children’s (e.g., Hendriks, 2016), is the result a consistent form-meaning mapping and hence a construction.

In addition to a production-comprehension asymmetry, the Optimality Theory account also predicts an effect of animacy on children’s comprehension of transitive sentences due to the competition between word order and animacy, namely that children perform best if the subject is animate and the object is inanimate. No interaction effect of subject animacy and object animacy was found in the picture selection task, which would have been in accordance with the stronger version of the relational animacy constraint that requires subjects to be higher in animacy than objects (e.g., de Swart, 2011; de Swart and van Bergen, 2019). However, children were more likely to select the correct interpretation when the subject was animate and additionally were less likely to select the correct interpretation when the object was animate. This is in accordance with the weaker version of the relational animacy constraint (cf. Aissen, 2003). Crucially, the results are not explained by the inherent animacy bias, which predicts that all animate entities are activated and retrieved more easily, and hence incorrectly predicts that animate direct objects should show a processing advantage compared to inanimate direct objects.

Animacy effects in children’s online comprehension in the preferential looking task were somewhat less pronounced but largely corroborate the offline findings, as a preference for animate subjects was found in the gaze patterns of the 3-year-olds (but not the 2-year-olds). The finding of animacy effects in offline and online comprehension thus confirms the results of earlier studies on Dutch and English that used an act-out methodology (on Dutch: Cannizzaro, 2012, Experiment 2; on English: Chapman and Miller, 1975; Thal and Flores, 2001; Chan et al., 2009; but see McClellan et al., 1986). The presence of animacy effects in both comprehension tasks provides evidence that children’s poor comprehension of transitive sentences is not caused by the demands of the experimental tasks used. This is unexpected from the perspective of generative approaches, which would have to explain the poor comprehension by task demands, but is compatible with usage-based approaches that consider animacy a heuristic in language use.

Interestingly, animacy effects were also present in adults’ response accuracy, RTs and gaze patterns in the picture selection task: adults were less likely to choose the subject-object interpretation, were slower to respond, and looked toward the picture reflecting this interpretation less strongly, when the subject was inanimate. This finding supports the view that animacy is not merely a heuristic that children rely on because of insufficient linguistic knowledge, but rather is a constraint of the adult grammar, albeit a weak constraint in Dutch that is generally overridden by the stronger word order constraint. It is also consistent with interactive sentence processing models in which animacy is considered an integral part of the form-to-meaning mapping, that is functionally equivalent to syntactic information such as word order (e.g., Bornkessel-Schlesewsky and Schlesewsky, 2009).

Not only did we find the predicted effects of animacy in comprehension, but we also found effects of animacy on children’s produced forms – but not on their gaze patterns – in the sentence elicitation task, that were not predicted by the Optimality Theory account. Although the children produced sentences with subject-object word order in over 80% of cases, they were more likely to do so when the subject was animate. The effect of animacy on children’s produced utterances could be due to the inherent animacy bias, which is argued to facilitate retrieval of animate entities from memory in sentence production (Bock and Warren, 1985; Branigan et al., 2008). If true, animacy has distinct effects in comprehension and production and competes with word order in children’s comprehension of transitive sentences, giving rise to poor understanding, but has a facilitating effect in children’s production of transitive sentences regardless of word order, leading to adult-like utterances.

In addition to effects of animacy, we also found effects of test verb, direction of action and side of the target picture. In the sentence elicitation task, children were more likely to produce subject-object order when the verb was *push* than when the verb was *pull*. Since the pre-test showed that the children could name the actions of pushing and pulling, it seems unlikely that this effect is caused by children’s weaker knowledge of the verb *pull*. Possibly, the action of pulling may have been less salient in the pictures compared to the action of pushing, as the action of pulling could only be identified by seeing the rope between the puller and the one being pulled. This explanation is supported by the similar looking behavior of adults and children: they all looked more to the agent when the verb was *pull*, although for adults this looking pattern was limited to the first time window, consistent with their overall faster processing. In the same task, the children, but not the adults, were also more likely to produce subject-object order when the direction of the action was to the left. In addition, the adults in the picture selection task and the 2-year-olds but not the 3-year-olds in the preferential looking task were more likely to look at the target picture if it was on the left, although for adults this effect decreased over the course of the trial. It is not obvious how these effects could be related to our experimental materials, as direction of action and side of the target picture were balanced across conditions. Possibly, the preference of adults for pictures on the left is related to the left-first response bias observed by Koranda et al. (2020) for adults in the action domain, which they suggest could be due to the fact that reading in English (and therefore also in Dutch) leads to eye fixations being ordered from left to right. Because Dutch parents and their young children read picture books from left to right too, this could also explain the 2-year-olds’ preference, which however did not surface in the 3-year-olds.

A limitation of this study is the severe data loss in the sentence elicitation task, in particular for the 2-year-olds. They produced a large number of unscorable utterances, which is not uncommon in sentence elicitation tasks with 2-year-olds that aim to elicit syntactic as opposed to lexical data (see, e.g., Chapman and Miller, 1975; Verhagen and Blom, 2014). Necessarily, the analyses of the produced utterances and gaze data of the 2-year-olds in the sentence elicitation task should be interpreted with caution. But note that the general pattern of elicited production of our 2-year-olds appears to be in line with observations about spontaneous production at this age: although Dutch 2-year-olds still frequently omit subjects and objects, they already correctly use word order in their spontaneous speech (e.g., de Haan and Tuijnman, 1988).

This is the first study comparing offline and online comprehension and production of transitive sentences in the same Dutch-speaking children as well as adult controls. As we pointed out in Section “Background,” the majority of main clauses in Dutch have SVO word order. The remaining main clauses in Dutch have other word orders, including OVS order. The accuracy results of the picture selection task confirm that Dutch-speaking adults interpret transitive sentences in isolation as SVO, as the adults in our study gave subject-object interpretations to the sentences they heard in 97% of cases. The offline and online results of the picture selection task and the preferential looking task additionally show that Dutch-speaking 2- and 3-year-old children still have difficulty using SVO word order consistently in their interpretations and are influenced by the animacy of subject and object.

In this study, we only looked at transitive sentences in isolation containing two definite full noun phrases. However, in natural conversations, features of the discourse context such as topicality or accessibility and formal and semantic features of the subject and object noun phrases such as definiteness or anaphoricity also play a role and may license word orders other than canonical SVO order. These context-dependent features of Dutch word order are expected to interact with canonical word order and animacy and to be acquired later. More research is needed to chart the developmental path of the use of these features in the production and comprehension of canonical and non-canonical word orders in the acquisition of Dutch.

## Data Availability Statement

The raw data supporting the conclusions of this article will be made available by the authors, without undue reservation.

## Ethics Statement

This study involving human participants was reviewed and approved by Commissie Ethische Toetsing Onderzoek (CETO), University of Groningen. Written informed consent to participate in this study was provided by the participants’ legal guardian/next of kin.

## Author Contributions

GC and PH contributed to the conception and design of the study, developed the coding guidelines, interpreted the results, and wrote the manuscript. GC carried out the experiment and performed the statistical analysis. Both authors read and approved the submitted version.

## Conflict of Interest

The authors declare that the research was conducted in the absence of any commercial or financial relationships that could be construed as a potential conflict of interest.
